# Redetermination of ethyl (3a-*cis*)-3a,8b-dihydr­oxy-2-methyl-4-oxo-3a,8b-dihydro-4*H*-indeno[1,2-*b*]furan-3-carboxyl­ate monohydrate

**DOI:** 10.1107/S1600536809039403

**Published:** 2009-10-03

**Authors:** P. S. Pereira Silva, Raza Murad Ghalib, Sayed Hasan Mehdi, Rokiah Hashim, Othman Sulaiman

**Affiliations:** a CEMDRX, Physics Department, University of Coimbra, P-3004-516 Coimbra, Portugal; b School of Industrial Technology, Universiti Sains Malaysia, 11800 Pulau Pinang, Malaysia

## Abstract

The crystal structure of the title compound, C_15_H_14_O_6_·H_2_O, has been redetermined from single-crystal X-ray data. The structure was originally determined by Peet *et al.* [*J. Heterocycl. Chem.* (1995), **32**, 33–41] but the atomic coordinates were not reported or deposited in the Cambridge Structural Database. The ethyl substituent is disordered over two sites with refined occupancies of 0.815 (6) and 0.185 (6). The indeno group is almost planar [maximum deviation 0.0922 (14) Å] and makes an angle of 68.81 (4)° with the furan ring. The fused ring molecules are assembled in pairs by intermolecular O—H⋯O hydrogen bonds. The resulting dimers are also hydrogen bonded to the water molecules, forming double-stranded chains running along the *a* axis.

## Related literature

For the previous report of the crystal structure, see: Peet *et al.* (1995 ▶). For chemical background, see: Black *et al.* (1994 ▶); Ruhemann (1910 ▶); Kaiser *et al.* (1970 ▶).
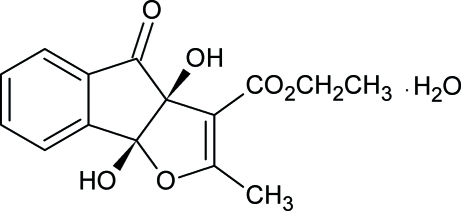


## Experimental

### 

#### Crystal data

C_15_H_14_O_6_·H_2_O
*M* *_r_* = 308.28
Monoclinic, 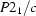

*a* = 7.9740 (4) Å
*b* = 16.7524 (8) Å
*c* = 11.0041 (5) Å
β = 98.833 (2)°
*V* = 1452.53 (12) Å^3^
*Z* = 4
Mo *K*α radiation
μ = 0.11 mm^−1^
*T* = 293 K
0.49 × 0.46 × 0.22 mm

#### Data collection

Bruker APEXII CCD area-detector diffractometer
Absorption correction: multi-scan (*SADABS*; Sheldrick, 2003 ▶) *T*_min_ = 0.894, *T*_max_ = 0.975
21608 measured reflections
3509 independent reflections
2820 reflections with *I* > 2σ(*I*)
*R*_int_ = 0.019

#### Refinement

*R*[*F* ^2^ > 2σ(*F* ^2^)] = 0.055
*wR*(*F* ^2^) = 0.154
*S* = 1.03
3509 reflections
215 parameters
27 restraints
H atoms treated by a mixture of independent and constrained refinement
Δρ_max_ = 0.50 e Å^−3^
Δρ_min_ = −0.38 e Å^−3^

### 

Data collection: *APEX2* (Bruker, 2003 ▶); cell refinement: *SAINT* (Bruker, 2003 ▶); data reduction: *SAINT*; program(s) used to solve structure: *SHELXS97* (Sheldrick, 2008 ▶); program(s) used to refine structure: *SHELXL97* (Sheldrick, 2008 ▶); molecular graphics: *PLATON* (Spek, 2009 ▶); software used to prepare material for publication: *SHELXL97*.

## Supplementary Material

Crystal structure: contains datablocks global, I. DOI: 10.1107/S1600536809039403/bt5075sup1.cif

Structure factors: contains datablocks I. DOI: 10.1107/S1600536809039403/bt5075Isup2.hkl

Additional supplementary materials:  crystallographic information; 3D view; checkCIF report

## Figures and Tables

**Table 1:** Hydrogen-bond geometry (Å, °)

*D*—H⋯*A*	*D*—H	H⋯*A*	*D*⋯*A*	*D*—H⋯*A*
O14—H14⋯O21^i^	0.82	1.90	2.7103 (17)	172
O21—H21⋯O22	0.82	1.94	2.724 (2)	159
O22—H22*A*⋯O14^ii^	0.83 (3)	2.45 (3)	3.124 (2)	140 (3)
O22—H22*B*⋯O17^iii^	0.89 (3)	2.11 (3)	2.972 (3)	163 (3)

Symmetry codes: (i) 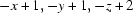; (ii) 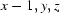; (iii) 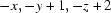.

**Fig. 1.**
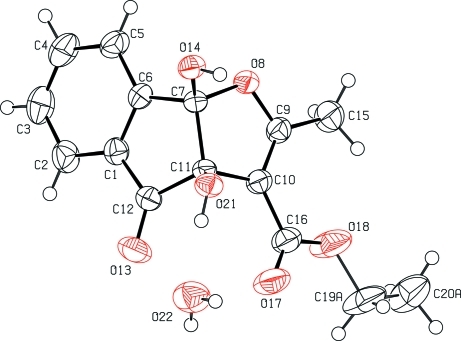
ORTEPII (Spek, 2009) plot of the title compound. Displacement ellipsoids are drawn at the 50% probability level. For clarity only the ethyl group with the highest occupancy is shown.

**Fig. 2.**
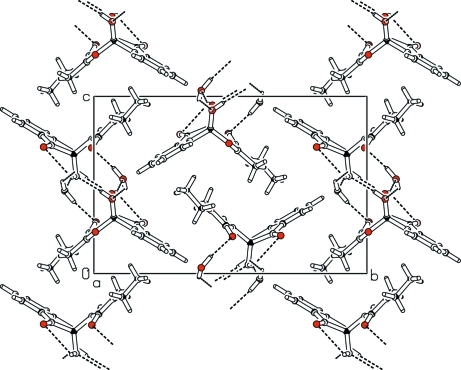
Packing diagram, viewed down the a axis, with the hydrogen bonds depicted as dashed lines.

## supplementary crystallographic information

### Comment

Ninhydrin (2,2-Dihydroxyindane-1,3-dione) is a chemical used to detect α-amino acids, proteins and dipeptides. When it reacts with these free amines, a deep blue or purple color known as Ruhemann's purple is evolved (Ruhemann, 1910; Kaiser *et al.*, 1970). It is one of the most widely used reagent for chemical development of fingerprints on porous surfaces. Ninhydrin in benzene undergoes electrophilic substitution at C2 of 3,5-dimethoxyaniline leading to the formation of indeno[1,2-*b*]indole. The corresponding reaction in water undergoes electrophilic substitution at C4 (Black *et al.*, 1994).

The ethyl substituent of the tile compound is disordered over two sites with refined occupancies of 0.815 (6) and 0.185 (6). The indeno moiety is almost planar, with atoms C11 and C12 deviating by -0.0574 (13) and 0.0922 (14) Å, respectively, from the indeno plane. The angle between the indeno group and the furan ring is 68.81 (4) °.

### Experimental

A mixture of ninhydrin (1.78 g) and ethyl acetoacetate (1.27 ml) in molar ratio 1:1 were refluxed in acetone for thirty minutes in presence of Mg/HCl. The reaction mixture was filtered and dried at low pressure. The dried mass was crystallized with solvent system diethyl ether and hexane to give transparent crystals (mp 373–376 K) of title compound (2.68 g).

The melting point was determined on a Kofler block melting point apparatus and is uncorrected.

### Refinement

Hydrogen atoms not belonging to the water molecule were placed at calculated positions and refined as riding on their parent atoms, using *SHELXL97* (Sheldrick, 2008) defaults [C—H = 0.93 Å, N—H = 0.86 Å and *U*_iso_(H) = 1.2*U*_eq_(C,N)]. The hydrogen atoms of the water molecule were included in the refinement riding on the O atom with *U*_iso_ = 1.5*U*_eq_(O).

We have chosen to model the positional disorder of the ethyl substituint with two groups. The ethyl C atoms were refined anisotropically with the *U_ij_* values restrained to behave   isotropically, with the ISOR instruction, and each C atom of one group was given the same displacement parameters as the corresponding atom of the other group with EADP instructions. The geometries of the two groups were made equivalent with SADI intructions.

### Crystal data

**Table:**

C_15_H_14_O_6_·H_2_O	*F*(000) = 648
*M**_r_* = 308.28	*D*_x_ = 1.410 Mg m^−^^3^
Monoclinic, *P*2_1_/*c*	Melting point = 373–376 K
Hall symbol: -P 2ybc	Mo *K*α radiation, λ = 0.71073 Å
*a* = 7.9740 (4) Å	Cell parameters from 5159 reflections
*b* = 16.7524 (8) Å	θ = 2.2–27.9°
*c* = 11.0041 (5) Å	µ = 0.11 mm^−^^1^
β = 98.833 (2)°	*T* = 293 K
*V* = 1452.53 (12) Å^3^	Parallelepipedic, colourless
*Z* = 4	0.49 × 0.46 × 0.22 mm

### Data collection

**Table:**

Bruker APEXII CCD area-detector diffractometer	3509 independent reflections
Radiation source: fine-focus sealed tube	2820 reflections with *I* > 2σ(*I*)
graphite	*R*_int_ = 0.019
φ and ω scans	θ_max_ = 28.0°, θ_min_ = 2.2°
Absorption correction: multi-scan (*SADABS*; Sheldrick, 2003)	*h* = −10→10
*T*_min_ = 0.894, *T*_max_ = 0.975	*k* = −22→22
21608 measured reflections	*l* = −9→14

### Refinement

**Table:**

Refinement on *F*^2^	Primary atom site location: structure-invariant direct methods
Least-squares matrix: full	Secondary atom site location: difference Fourier map
*R*[*F*^2^ > 2σ(*F*^2^)] = 0.055	Hydrogen site location: inferred from neighbouring sites
*wR*(*F*^2^) = 0.154	H atoms treated by a mixture of independent and constrained refinement
*S* = 1.03	*w* = 1/[σ^2^(*F*_o_^2^) + (0.0682*P*)^2^ + 0.9584*P*] where *P* = (*F*_o_^2^ + 2*F*_c_^2^)/3
3509 reflections	(Δ/σ)_max_ < 0.001
215 parameters	Δρ_max_ = 0.50 e Å^−^^3^
27 restraints	Δρ_min_ = −0.38 e Å^−^^3^

### Special details

**Table:**

Geometry. All e.s.d.'s (except the e.s.d. in the dihedral angle between two l.s. planes) are estimated using the full covariance matrix. The cell e.s.d.'s are taken into account individually in the estimation of e.s.d.'s in distances, angles and torsion angles; correlations between e.s.d.'s in cell parameters are only used when they are defined by crystal symmetry. An approximate (isotropic) treatment of cell e.s.d.'s is used for estimating e.s.d.'s involving l.s. planes.
Refinement. Refinement of *F*^2^ against ALL reflections. The weighted *R*-factor *wR* and goodness of fit *S* are based on *F*^2^, conventional *R*-factors *R* are based on *F*, with *F* set to zero for negative *F*^2^. The threshold expression of *F*^2^ > σ(*F*^2^) is used only for calculating *R*-factors(gt) *etc*. and is not relevant to the choice of reflections for refinement. *R*-factors based on *F*^2^ are statistically about twice as large as those based on *F*, and *R*- factors based on ALL data will be even larger.

### Fractional atomic coordinates and isotropic or equivalent isotropic displacement parameters (Å^2^)

**Table:**

	*x*	*y*	*z*	*U*_iso_*/*U*_eq_	Occ. (<1)
O8	0.60398 (14)	0.49466 (7)	0.73529 (11)	0.0366 (3)	
O13	0.19037 (18)	0.31575 (9)	0.78551 (16)	0.0590 (4)	
O14	0.69530 (14)	0.43316 (7)	0.92132 (11)	0.0353 (3)	
H14	0.6687	0.4710	0.9617	0.053*	
O17	0.04534 (18)	0.49231 (10)	0.79040 (16)	0.0641 (5)	
O18	0.10479 (19)	0.57149 (12)	0.64190 (19)	0.0820 (6)	
O21	0.36289 (15)	0.43437 (7)	0.95007 (10)	0.0354 (3)	
H21	0.2683	0.4176	0.9572	0.053*	
C1	0.4633 (2)	0.30757 (10)	0.71894 (16)	0.0368 (4)	
C2	0.4601 (3)	0.23627 (12)	0.65306 (19)	0.0488 (5)	
H2	0.3606	0.2069	0.6338	0.059*	
C3	0.6090 (3)	0.21116 (13)	0.6179 (2)	0.0568 (5)	
H3	0.6102	0.1642	0.5729	0.068*	
C4	0.7575 (3)	0.25430 (13)	0.6479 (2)	0.0552 (5)	
H4	0.8572	0.2349	0.6247	0.066*	
C5	0.7612 (2)	0.32559 (12)	0.71189 (18)	0.0443 (4)	
H5	0.8609	0.3549	0.7308	0.053*	
C6	0.6107 (2)	0.35153 (10)	0.74655 (15)	0.0331 (3)	
C7	0.58235 (19)	0.42670 (9)	0.81511 (15)	0.0298 (3)	
C9	0.4506 (2)	0.52675 (10)	0.69420 (16)	0.0353 (4)	
C10	0.3220 (2)	0.49272 (10)	0.74201 (15)	0.0349 (4)	
C11	0.38915 (19)	0.42608 (9)	0.82712 (14)	0.0295 (3)	
C12	0.3265 (2)	0.34408 (10)	0.77556 (16)	0.0364 (4)	
C15	0.4550 (3)	0.59275 (13)	0.6050 (2)	0.0521 (5)	
H15A	0.4636	0.5710	0.5255	0.078*	
H15B	0.5514	0.6262	0.6316	0.078*	
H15C	0.3530	0.6237	0.6003	0.078*	
C16	0.1456 (2)	0.51738 (12)	0.72845 (19)	0.0473 (5)	
C19A	−0.0681 (3)	0.6050 (2)	0.6333 (4)	0.0752 (11)	0.815 (6)
H19A	−0.1528	0.5633	0.6168	0.090*	0.815 (6)
H19B	−0.0826	0.6318	0.7092	0.090*	0.815 (6)
C20A	−0.0827 (4)	0.6619 (2)	0.5313 (4)	0.0808 (11)	0.815 (6)
H20A	−0.1885	0.6900	0.5258	0.121*	0.815 (6)
H20B	−0.0787	0.6336	0.4559	0.121*	0.815 (6)
H22C	0.0094	0.6993	0.5452	0.121*	0.815 (6)
C19B	−0.0660 (10)	0.5823 (6)	0.5692 (13)	0.0752 (11)	0.185 (6)
H19C	−0.0656	0.5693	0.4833	0.090*	0.185 (6)
H19D	−0.1491	0.5491	0.6008	0.090*	0.185 (6)
C20B	−0.1022 (16)	0.6673 (7)	0.5842 (16)	0.0808 (11)	0.185 (6)
H20D	−0.2188	0.6779	0.5520	0.121*	0.185 (6)
H20E	−0.0302	0.6988	0.5406	0.121*	0.185 (6)
H20F	−0.0811	0.6809	0.6700	0.121*	0.185 (6)
O22	0.0700 (2)	0.39329 (12)	1.03321 (17)	0.0620 (4)	
H22A	−0.014 (4)	0.385 (2)	0.981 (3)	0.093*	
H22B	0.057 (4)	0.4267 (19)	1.094 (3)	0.093*	

### Atomic displacement parameters (Å^2^)

**Table:**

	*U*^11^	*U*^22^	*U*^33^	*U*^12^	*U*^13^	*U*^23^
O8	0.0297 (6)	0.0356 (6)	0.0467 (7)	0.0007 (5)	0.0130 (5)	0.0072 (5)
O13	0.0427 (7)	0.0543 (8)	0.0839 (11)	−0.0176 (6)	0.0219 (7)	−0.0161 (8)
O14	0.0289 (5)	0.0400 (6)	0.0368 (6)	0.0009 (5)	0.0037 (5)	−0.0057 (5)
O17	0.0353 (7)	0.0791 (11)	0.0817 (11)	0.0117 (7)	0.0211 (7)	0.0296 (9)
O18	0.0373 (8)	0.1084 (14)	0.1015 (14)	0.0201 (8)	0.0146 (8)	0.0643 (11)
O21	0.0326 (6)	0.0419 (6)	0.0340 (6)	−0.0026 (5)	0.0124 (5)	−0.0021 (5)
C1	0.0393 (9)	0.0326 (8)	0.0386 (8)	0.0005 (7)	0.0060 (7)	−0.0035 (7)
C2	0.0557 (11)	0.0380 (9)	0.0515 (11)	−0.0015 (8)	0.0048 (9)	−0.0099 (8)
C3	0.0738 (14)	0.0420 (10)	0.0554 (12)	0.0103 (10)	0.0124 (10)	−0.0158 (9)
C4	0.0573 (12)	0.0542 (12)	0.0580 (12)	0.0178 (10)	0.0211 (10)	−0.0093 (10)
C5	0.0384 (9)	0.0474 (10)	0.0491 (10)	0.0074 (8)	0.0131 (8)	−0.0057 (8)
C6	0.0335 (8)	0.0329 (8)	0.0334 (8)	0.0041 (6)	0.0063 (6)	−0.0015 (6)
C7	0.0267 (7)	0.0301 (7)	0.0336 (7)	0.0002 (6)	0.0078 (6)	0.0002 (6)
C9	0.0356 (8)	0.0340 (8)	0.0374 (8)	0.0035 (6)	0.0096 (6)	0.0020 (6)
C10	0.0308 (8)	0.0367 (8)	0.0377 (8)	0.0023 (6)	0.0070 (6)	0.0026 (7)
C11	0.0253 (7)	0.0312 (7)	0.0330 (7)	−0.0005 (6)	0.0075 (6)	−0.0011 (6)
C12	0.0323 (8)	0.0347 (8)	0.0423 (9)	−0.0032 (6)	0.0067 (7)	−0.0027 (7)
C15	0.0534 (11)	0.0497 (11)	0.0564 (12)	0.0053 (9)	0.0182 (9)	0.0179 (9)
C16	0.0333 (9)	0.0512 (11)	0.0577 (11)	0.0062 (8)	0.0080 (8)	0.0149 (9)
C19A	0.0372 (12)	0.114 (3)	0.076 (2)	0.0281 (14)	0.0150 (14)	0.043 (2)
C20A	0.0680 (18)	0.102 (2)	0.075 (3)	0.0325 (17)	0.0190 (17)	0.028 (2)
C19B	0.0372 (12)	0.114 (3)	0.076 (2)	0.0281 (14)	0.0150 (14)	0.043 (2)
C20B	0.0680 (18)	0.102 (2)	0.075 (3)	0.0325 (17)	0.0190 (17)	0.028 (2)
O22	0.0539 (9)	0.0752 (11)	0.0602 (10)	−0.0025 (8)	0.0193 (7)	0.0090 (8)

### Geometric parameters (Å, °)

**Table:**

O8—C9	1.349 (2)	C7—C11	1.566 (2)
O8—C7	1.4643 (19)	C9—C10	1.349 (2)
O13—C12	1.205 (2)	C9—C15	1.482 (3)
O14—C7	1.3658 (19)	C10—C16	1.452 (2)
O14—H14	0.8200	C10—C11	1.502 (2)
O17—C16	1.203 (2)	C11—C12	1.540 (2)
O18—C16	1.319 (2)	C15—H15A	0.9600
O18—C19A	1.478 (3)	C15—H15B	0.9600
O18—C19B	1.481 (5)	C15—H15C	0.9600
O21—C11	1.4073 (19)	C19A—C20A	1.463 (4)
O21—H21	0.8200	C19A—H19A	0.9700
C1—C6	1.380 (2)	C19A—H19B	0.9700
C1—C2	1.395 (2)	C20A—H20A	0.9600
C1—C12	1.470 (2)	C20A—H20B	0.9600
C2—C3	1.370 (3)	C20A—H22C	0.9600
C2—H2	0.9300	C19B—C20B	1.468 (6)
C3—C4	1.384 (3)	C19B—H19C	0.9700
C3—H3	0.9300	C19B—H19D	0.9700
C4—C5	1.384 (3)	C20B—H20D	0.9600
C4—H4	0.9300	C20B—H20E	0.9600
C5—C6	1.384 (2)	C20B—H20F	0.9600
C5—H5	0.9300	O22—H22A	0.83 (3)
C6—C7	1.503 (2)	O22—H22B	0.89 (3)
			
C9—O8—C7	109.07 (12)	C10—C11—C7	101.73 (12)
C7—O14—H14	109.5	C12—C11—C7	104.03 (12)
C16—O18—C19A	115.58 (18)	O13—C12—C1	127.76 (16)
C16—O18—C19B	125.4 (6)	O13—C12—C11	124.31 (16)
C19A—O18—C19B	31.6 (5)	C1—C12—C11	107.89 (13)
C11—O21—H21	109.5	C9—C15—H15A	109.5
C6—C1—C2	121.21 (17)	C9—C15—H15B	109.5
C6—C1—C12	110.30 (15)	H15A—C15—H15B	109.5
C2—C1—C12	128.35 (17)	C9—C15—H15C	109.5
C3—C2—C1	117.51 (19)	H15A—C15—H15C	109.5
C3—C2—H2	121.2	H15B—C15—H15C	109.5
C1—C2—H2	121.2	O17—C16—O18	122.50 (18)
C2—C3—C4	121.28 (18)	O17—C16—C10	124.07 (18)
C2—C3—H3	119.4	O18—C16—C10	113.43 (16)
C4—C3—H3	119.4	C20A—C19A—O18	105.1 (2)
C3—C4—C5	121.52 (19)	C20A—C19A—H19A	110.7
C3—C4—H4	119.2	O18—C19A—H19A	110.7
C5—C4—H4	119.2	C20A—C19A—H19B	110.7
C6—C5—C4	117.34 (18)	O18—C19A—H19B	110.7
C6—C5—H5	121.3	H19A—C19A—H19B	108.8
C4—C5—H5	121.3	C19A—C20A—H20A	109.5
C1—C6—C5	121.12 (16)	C19A—C20A—H20B	109.5
C1—C6—C7	111.65 (14)	H20A—C20A—H20B	109.5
C5—C6—C7	127.23 (16)	C19A—C20A—H22C	109.5
O14—C7—O8	109.19 (12)	H20A—C20A—H22C	109.5
O14—C7—C6	111.45 (13)	H20B—C20A—H22C	109.5
O8—C7—C6	108.04 (13)	C20B—C19B—O18	103.8 (5)
O14—C7—C11	117.14 (13)	C20B—C19B—H19C	111.0
O8—C7—C11	105.29 (12)	O18—C19B—H19C	111.0
C6—C7—C11	105.22 (12)	C20B—C19B—H19D	111.0
O8—C9—C10	113.90 (15)	O18—C19B—H19D	111.0
O8—C9—C15	113.99 (15)	H19C—C19B—H19D	109.0
C10—C9—C15	132.10 (17)	C19B—C20B—H20D	109.5
C9—C10—C16	128.49 (16)	C19B—C20B—H20E	109.5
C9—C10—C11	109.76 (14)	H20D—C20B—H20E	109.5
C16—C10—C11	121.49 (15)	C19B—C20B—H20F	109.5
O21—C11—C10	115.77 (13)	H20D—C20B—H20F	109.5
O21—C11—C12	110.76 (13)	H20E—C20B—H20F	109.5
C10—C11—C12	111.65 (13)	H22A—O22—H22B	117 (3)
O21—C11—C7	111.96 (12)		
			
C6—C1—C2—C3	0.6 (3)	O14—C7—C11—O21	1.34 (19)
C12—C1—C2—C3	−174.61 (19)	O8—C7—C11—O21	−120.20 (13)
C1—C2—C3—C4	0.9 (3)	C6—C7—C11—O21	125.78 (13)
C2—C3—C4—C5	−1.7 (4)	O14—C7—C11—C10	125.58 (14)
C3—C4—C5—C6	1.1 (3)	O8—C7—C11—C10	4.03 (15)
C2—C1—C6—C5	−1.3 (3)	C6—C7—C11—C10	−109.99 (14)
C12—C1—C6—C5	174.74 (16)	O14—C7—C11—C12	−118.30 (14)
C2—C1—C6—C7	178.60 (16)	O8—C7—C11—C12	120.15 (13)
C12—C1—C6—C7	−5.4 (2)	C6—C7—C11—C12	6.13 (16)
C4—C5—C6—C1	0.4 (3)	C6—C1—C12—O13	−168.21 (19)
C4—C5—C6—C7	−179.43 (17)	C2—C1—C12—O13	7.4 (3)
C9—O8—C7—O14	−131.60 (14)	C6—C1—C12—C11	9.40 (19)
C9—O8—C7—C6	107.02 (14)	C2—C1—C12—C11	−174.95 (18)
C9—O8—C7—C11	−5.02 (16)	O21—C11—C12—O13	47.9 (2)
C1—C6—C7—O14	127.18 (15)	C10—C11—C12—O13	−82.7 (2)
C5—C6—C7—O14	−52.9 (2)	C7—C11—C12—O13	168.40 (18)
C1—C6—C7—O8	−112.85 (15)	O21—C11—C12—C1	−129.76 (14)
C5—C6—C7—O8	67.0 (2)	C10—C11—C12—C1	99.64 (15)
C1—C6—C7—C11	−0.76 (18)	C7—C11—C12—C1	−9.31 (17)
C5—C6—C7—C11	179.11 (17)	C19A—O18—C16—O17	5.4 (4)
C7—O8—C9—C10	4.1 (2)	C19B—O18—C16—O17	−29.6 (7)
C7—O8—C9—C15	−175.85 (15)	C19A—O18—C16—C10	−173.7 (3)
O8—C9—C10—C16	172.92 (18)	C19B—O18—C16—C10	151.3 (7)
C15—C9—C10—C16	−7.1 (3)	C9—C10—C16—O17	−166.7 (2)
O8—C9—C10—C11	−1.3 (2)	C11—C10—C16—O17	6.9 (3)
C15—C9—C10—C11	178.71 (19)	C9—C10—C16—O18	12.3 (3)
C9—C10—C11—O21	119.75 (16)	C11—C10—C16—O18	−174.08 (18)
C16—C10—C11—O21	−54.9 (2)	C16—O18—C19A—C20A	−179.3 (3)
C9—C10—C11—C12	−112.29 (16)	C19B—O18—C19A—C20A	−62.8 (10)
C16—C10—C11—C12	73.0 (2)	C16—O18—C19B—C20B	127.2 (10)
C9—C10—C11—C7	−1.87 (18)	C19A—O18—C19B—C20B	45.5 (9)
C16—C10—C11—C7	−176.54 (16)		

### Hydrogen-bond geometry (Å, °)

**Table:**

*D*—H···*A*	*D*—H	H···*A*	*D*···*A*	*D*—H···*A*
O14—H14···O21^i^	0.82	1.90	2.7103 (17)	172
O21—H21···O22	0.82	1.94	2.724 (2)	159
O22—H22A···O14^ii^	0.83 (3)	2.45 (3)	3.124 (2)	140 (3)
O22—H22B···O17^iii^	0.89 (3)	2.11 (3)	2.972 (3)	163 (3)

Symmetry codes: (i) −*x*+1, −*y*+1, −*z*+2; (ii) *x*−1, *y*, *z*; (iii) −*x*, −*y*+1, −*z*+2.
